# Assessing sexual dimorphism in the common vampire bat, *Desmodus rotundus*

**DOI:** 10.1371/journal.pone.0320169

**Published:** 2026-01-21

**Authors:** Analorena Cifuentes-Rincon, Karen D. Sarmiento-Arias, Diego Soler-Tovar, Abelardo Rodríguez-Bolaños, Carlos Bravo-Garcia, Nicolas Reyes-Amaya, Laura Ávila-Vargas, Luis E. Escobar

**Affiliations:** 1 Department of Fish and Wildlife Conservation, College of Natural Resources and Environment, Virginia Tech, Blacksburg, Virginia, United States of America; 2 Laboratorio de Biodiversidad, Instituto de Ciencias, Benemérita Universidad Autónoma de Puebla, Puebla, México; 3 Epidemiology and Public Health Research Group, Faculty of Agricultural Sciences, Universidad de La Salle, Bogotá, Colombia; 4 Programa de Biología, Facultad de Ciencias Matemáticas y Naturales, Grupo de Investigación Biodiversidad de Alta Montana, Museo de Historia Natural, Universidad Distrital Francisco José de Caldas, Bogotá, Colombia; 5 Colección de mamíferos, Instituto de Investigación de Recursos Biológicos Alexander von Humboldt, Villa de Leyva, Boyacá, Colombia; 6 Center for Emerging, Zoonotic, and Arthropod-borne Pathogens, Virginia Tech, Blacksburg, Virginia, United States of America; 7 One Health Institute, Faculty of Life Sciences, Universidad Andres Bello, Santiago, Chile; 8 Global Change Center, Virginia Tech, Blacksburg, Virginia, United States of America; Sao Paulo State University Julio de Mesquita Filho: Universidade Estadual Paulista Julio de Mesquita Filho, BRAZIL

## Abstract

Sexual dimorphism in bats is understudied, with conflicting evidence across species and geographic regions. For *Desmodus rotundus*, the common vampire bat, previous reports on morphological sex differences have been inconsistent. This study aimed to assess sexual dimorphism in *D. rotundus* using a combination of contemporary field measurements and historical museum specimens. We analyzed six morphometric traits, including body mass, head length, body length, tibia length, ear length, and forearm length. Data were collected from 46 wild-captured individuals from five locations across Colombia in South America. Additionally, forearm length was examined in an expanded dataset of 490 specimens, including additional 444 individuals from museum vouchers collected over the past century. Principal components analysis and hierarchical clustering of the six-trait dataset showed patterns of differentiation between sexes, with partial overlap. Forearm length, analyzed independently in the full 490-specimen dataset, showed strong evidence of sexual dimorphism. Females had significantly longer forearms (mean = 61.8 mm) than males (mean = 58.5 mm), with non-overlapping 95% confidence intervals and a highly significant t-test result (t = −12.68, p < 2 × 10 ⁻ ¹⁶). Sex explained 25.7% of the variation in forearm length (R² = 0.26). Tibia length also differed significantly between sexes of the wild-catch individuals (p = 0.004), with females exhibiting greater values. Comparisons between museum specimens (historical) and wild-caught specimens (contemporary) showed no significant differences across time in either sex. Among females, the difference was not significant (t = −0.93, df = 208, p = 0.355), and the same was true for males (t = −0.01, df = 278, p = 0.992). A follow-up MANOVA on the six morphometric traits indicated a significant effect of sex (Pillai’s trace = 0.389, approx. F(6,39)=4.14, p < 2.2 × 10 ⁻ ¹⁶). After correcting for multiple comparisons, significant sexual dimorphism remained for forearm and tibia lengths, with forearm showing the strongest signal. These findings provide robust support for modest but consistent female-biased dimorphism in *D. rotundus*. The use of both multivariate and univariate analysis, combined with long-term historical data, enhanced the reliability of signals detected regarding morphological differences. *Desmodus rotundus* play a role as a primary reservoir for zoonotic viruses, has potential relevance in biomedical research, and provides ecosystem services. Understanding sex-based morphological variation is critical to inform public health, ecology, and biological conservation strategies. Females were consistently larger than males, but segregation was not absolute, with some individuals falling outside the expected data range for their sex. This study contributes to a clearer understanding of morphological variation and lays the groundwork for future research into the ecological and evolutionary drivers of dimorphism in bats.

## Introduction

Latin America is home to the only sanguivorous bat species in the world: *Diphylla ecaudata* (Spix, 1823), *Diaemus youngi* (Jentink, 1893), and *Desmodus rotundus* (E. Geoffroy, 1810) [1]. Among sanguivorous bats, *D. rotundus* is unique for being the only extant member of its genus and for specializing in feeding on mammals [2,3]. Its distribution spans from northern Mexico through Central America and much of South America [4–6], occupying a variety of habitats, from sea level to elevations over 3600 meters [7], including deserts, savannas, and tropical forests [8–10]. Its ability to thrive in disturbed environments, especially areas with livestock, underscores its ecological plasticity [3,11,12].

*Desmodus rotundus* is also a highly social species, living in colonies typically composed of a dominant male and several females [13]. Its reproductive biology is flexible, with breeding occurring year-round but peaking during rainy seasons, when an influx of juveniles can reshape colony dynamics [14–16]. The species’ obligate diet of blood has long intrigued researchers and generated public concern, particularly in rural areas of Latin America where *D. rotundus* can transmit rabies virus and other zoonotic pathogens to different species [6,15,16]. As a result, *D. rotundus* is not only ecologically significant but also a focal species in public health and wildlife disease management efforts.

Beyond its importance to public health, *D. rotundus* has drawn scientific interest due to its potential for use in biomedical research across Neotropics [6,15,17,18]. Recent studies have identified bioactive compounds in *D. rotundus* saliva that show promise in the treatment of cardiovascular diseases, such as stroke and hypertension [16,17,19–24]. These discoveries reflect the importance of studying *D. rotundus* from a public health perspective, as an important species for ecosystem services, and as a source of medical innovation [25–27].

In *D. rotundus*, sexual differences extend beyond morphology to include reproductive roles, social behavior, and feeding strategies. Females are often more involved in social cohesion activities such as grooming and regurgitative food sharing [28,29], while males invest more in maintaining dominance and reproductive access within colonies [11,14]. While behavioral, immunological, and physiological aspects of *D. rotundus* have received considerable attention, its morphological variation, including sexual dimorphism, remains poorly understood.

Current literature on sexual dimorphism in *D. rotundus* presents conflicting results. For instance, some studies report that females are larger than males, especially in traits linked to body size [30–33]. Other studies, however, suggest that males exhibit larger traits due to sexual selection and competitive behaviors [34]. Additional reports find no significant sexual differences, suggesting that environmental variables such as food availability may play a more dominant role [35]. These inconsistencies highlight a gap in our understanding of sexual dimorphism in *D. rotundus* and the need for a broader, more geographically inclusive analysis.

In this study, we explore sexual dimorphism in *D. rotundus* using a comprehensive morphological dataset that includes recently collected specimens (2022–2023) and historical museum records spanning over a century (1921–2023) from 11 biological collections. We focus on populations from Colombia, a country that offers a wide range of ecological and elevational conditions, to investigate whether consistent patterns of dimorphism emerge. Our aim is to contribute to a more nuanced understanding of *D. rotundus*’ morphological variability and its relevance to evolutionary biology, species management, and biomedical studies.

## Materials and methods

**Fig 1:**
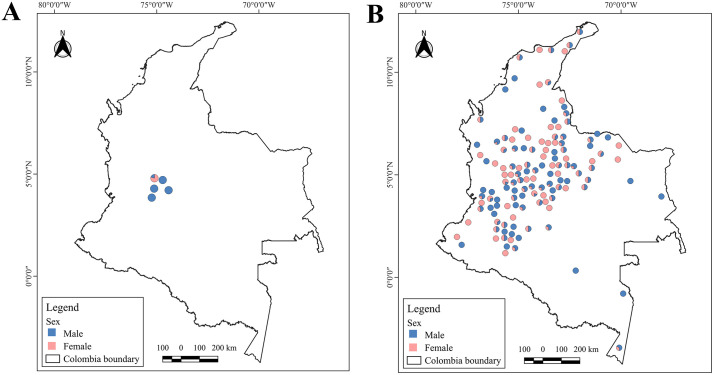
Geographical representation of the records of *Desmodus rotundus* in Colombia, represented by sex. **(A)** Contemporary dataset. Geographic distribution of 46 *D. rotundus* individuals from which six morphological traits were collected (i.e., weight, head length, body length, tibia length, ear length, and forearm length.) in the departments of Tolima and Cundinamarca. **(B)** Contemporary and historical dataset. Distribution of 490 *D. rotundus* museum specimens from which forearm measurements were collected for this study. Orange: female. Blue: male. Map was created using QGIS software/ Open source geospatial foundation project [36].

We collected morphometric data from two distinct data sets (contemporary and historical) to evaluate sexual dimorphism in *Desmodus rotundus*. The contemporary dataset comprised 46 live-captured individuals from fieldwork at five field sites from 2022 to 2023 across Colombia (Fig 1A). The historical dataset included 444 adult *D. rotundus* from museum specimens. Combined, we obtained data from 490 adult *D. rotundus*, including the 46 live individuals with 444 museum specimens.

### Ethics statement

Bat capture and sampling procedures were conducted in accordance with ethical guidelines for wildlife research and International Guiding Principles for Biomedical Research Involving Animals [37]. Protocols followed recommendations from the American Society of Mammalogists [38], and all methods were approved by the Virginia Tech Institutional Animal Care and Use Committee (IACUC-21–138) in the US, and Universidad de La Salle in Colombia under permit 1473 for the Collection of Specimens of Wild Species. Sampling to generate a contemporary dataset was carried out in compliance with national and international regulations for the ethical treatment of wild animals.

A veterinarian was present for all procedures to provide oversight and ensure appropriate handling, immobilization, anesthesia, euthanasia, and implementation of biosafety precautions to prevent human exposure to anesthetic agents [39].

### Bat capture and sampling methods

Bats were collected using three capture methods (i.e., conventional mist nets, harp trap, and cone trap) and placed in disinfected cloth bags [40,41]. To minimize stress and potential harm during processing, bats were handled gently and restrained carefully by trained personnel. Handling time was minimized (approximately 2 minutes per individual).

**Fig 2:**
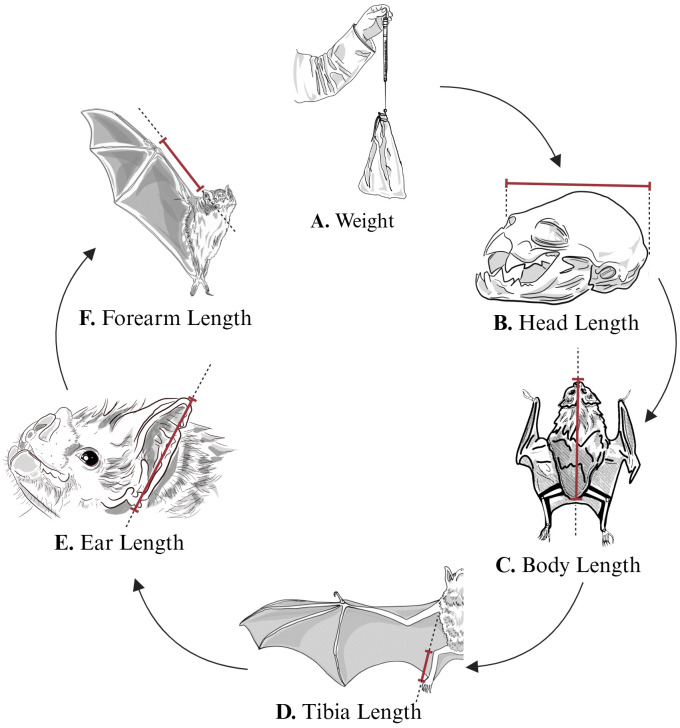
Morphometric measurements of *Desmodus rotundus.* Six key morphological traits were measured in individual bats: **(A)** Weight, measured using a digital balance, recorded in grams (g). **(B)** Head length, from the tip of the nasal leaf to the posterior edge of the skull (in mm). **(C)** Body length, from the tip of the nose to the base of the tail. **(D)** Tibia length, measured from the proximal end of the tibia to the posterior base of the calcar (in mm). **(E)** Ear length, from the base of the ear to the tip (in mm). **(F)** Forearm length, from the wrist to the elbow when the wing is folded (in mm). Red lines indicate the specific landmarks used for each measurement to visually demonstrate the precise locations.

We selected weight, head length, body length, tibia length, ear length, and forearm length as focal metrics to assess sexual dimorphism from the contemporary dataset (Fig 2). These measurements are standard in bat taxonomy and provide a robust source for exploring morphological variation [42–45]. Each metric was chosen based on its biological relevance, accuracy in differentiating sexes, and frequent use in bat studies [46].

A spring balance was used to weigh individuals, and the breeding stage was assessed. Adults were classified based on their reproductive condition (females were classified as active or inactive, lactating or pregnant, and males as having inguinal or descended testicles) [7,47]. Morphometric data were collected using a Uline digital caliper (accuracy: 0.0005 mm), and only adult and semi-adult individuals were included. Sexually immature or pregnant specimens were excluded to avoid confounding effects. Specimens were confirmed to be the species *D. rotundus* using broadly accepted taxonomic keys [7,47,48].

### Anesthesia and lethal sampling protocol

Specimens included in this study were obtained from individuals that had been lethally sampled as part of independent virological surveillance studies aimed at detecting rabies virus circulation in the study areas. Morphological measurements were taken prior to euthanasia, and no animals were euthanized specifically for the purpose of this morphological study.

For euthanasia, bats were gently placed inside a Ziploc-type plastic bag containing a cotton pad pre-soaked with 2 mL of USP-grade isoflurane, positioned in a corner to avoid direct contact with the animal. The bag was sealed immediately, and bats were monitored for signs of anesthesia (e.g., decreased responsiveness to stimuli, loss of righting reflex, and slowed respiration). Once deep anesthesia was confirmed (absence of reflexes), cardiac puncture was performed for exsanguination, followed by cervical dislocation to ensure death. Death was confirmed by the absence of heartbeat, respiration, and the presence of fixed, dilated pupils.

Organs were extracted for virological analyses, and voucher specimens were prepared and deposited at Colección de Mastozoología del Museo de La Salle – Bogotá, Colombia (MLS).

### Historical specimen collection

In bats, forearm length which inludes the segment of the wing extending from the elbow to the wrist, is the most reliable and widely used measurement to assess size [49,50]. This metric is diagnostically valuable due to its relevance to aerodynamics, ecological adaptation, geographic variation, and sexual dimorphism [51]. For this study, we obtained additional forearm length measurements from 444 specimens resulting in a total sample size of n = 490 (210 females and 280 males), including 444 historical specimens and contemporary bats (Fig 1B).

For museum specimen collection, we visited 11 museums in Colombia, including Colección de Mastozoología del Museo de La Salle (MLS-mam) [52], Colección de Mamíferos, Museo de Historia Natural C.J. Marinkelle, Universidad de los Andes (ANDES-M) [53], Colección de Mamíferos “Alberto Cadena García” Instituto de Ciencias Naturales, Universidad Nacional de Colombia (ICN) [54], Colección de Mamíferos del Instituto Humboldt (IAvH-M) [55], Colección de Mamíferos, Museo de Ciencias Naturales de La Salle del Instituto Tecnológico Metropolitano (CSJ-m) [56], Colección de Mamíferos del Museo de Historia Natural de la Universidad de la Amazonia (UAM-M), Colección Mastozoológica, Museo de Historia Natural Unillanos (MHNU-M) [57], Museo de Historia Natural de la Universidad Industrial de Santander (MHN-UIS), Colección Zoológica de la Universidad del Tolima (CZUT), Colección de Mamíferos, Museo de Historia Natural Universidad de (Caldas -Uca) [58], and Museo de Historia Natural de la Universidad Distrital Francisco José de Caldas (MHNUD-M) [59]. We measured the forearm length of specimens of *D. rotundus* from museum collections between October 2022 and April 2023. All forearm length measurements were taken using a 0–150 mm (6″) electronic digital caliper with an accuracy of 0.01 mm. Forearm data were employed alone to estimate sexual dimorphism from these and wild individuals across Colombia (Fig 1B).

### Data preprocessing and cleaning

All measurements were inspected for consistency, and individuals with missing or unclear sex assignment or forearm measurements were excluded. For the multivariate analysis, only the 46 live individuals with complete morphometric data were used. For the univariate analysis focused on forearm length, the complete sample of 490 individuals was analyzed.

### Statistical analyses

#### Principal component analysis.

We performed a Principal Component Analysis (PCA) to explore multivariate patterns of sexual dimorphism using six external morphological variables collected from the 46 contemporary individuals. Measurements were scaled and centered prior to analysis to ensure comparability. PCA was conducted using the ‘prcomp’ function in R v4.4.0, and results were visualized with the ggplot2 and factoextra packages [60]. The first two principal components were plotted to evaluate clustering by sex.

Finally, the correlation between ear and forearm, forearm and weight, and body and weight measurements were measured to corroborate the influence of the variables.

#### Univariate analyses.

We conducted two-sample Welch’s t-tests comparing males and females to assess sexual dimorphism in forearm length across the larger sample (n = 490). Prior to the test, we assessed data for normality (via Q-Q plots) and homogeneity of variances.

A simple linear regression model was used to assess the effect of sex on forearm length. The model was fitted using the ‘lm’ function in R, with sex as a categorical predictor and forearm length as the response variable. The significance of the predictor variable (sex) was evaluated using the associated p-value of the regression coefficient, with a threshold of α = 0.05. Model fit was assessed using the coefficient of determination (R²), and the residual standard error was used as an estimate of the variability in forearm length not explained by sex.

Comparisons of the six morphometric traits between sexes were conducted using Welch’s t-tests [61]. To account for multiple testing, p-values were adjusted using the Benjamini-Hochberg false discovery rate (FDR) procedure [62], with Bonferroni correction applied for comparison. Differences between sexes were further tested using a MANOVA including all six morphometric traits simultaneously [63].

#### Descriptive statistics.

We calculated sample size (n), mean (m), standard deviation (SD), interquartile range (IQR), and 95% confidence intervals (CI) for forearm length by sex using the dplyr and ggplot2 packages in R [60]. These descriptive statistics provided context for interpreting the degree and consistency of sexual dimorphism.

#### Hierarchical clustering.

Hierarchical clustering was performed using the dendextend package, ‘hclust’ function in R to further evaluate morphometric differentiation between sexes [64], on the scaled morphometric data using Euclidean distances and Ward’s method (ward.D2). Pearson correlation coefficients were calculated to assess associations among traits and selected pairwise correlations were tested using ‘cor.test’.

Two separate dendrograms were generated from the hierarchical clustering. One dendogram was based on the six scaled morphological variables from the 46 contemporary individuals, offering a multivariate perspective on sex-based clustering. The second dendogram was created using the scaled forearm length data for all 490 individuals, enabling visualization of sexual dimorphism in this specific trait across a broader sample. In both cases, the resulting dendrograms were annotated by sex to assess clustering patterns visually.

The hierarchical clustering was converted into a phylogenetic tree through the ape package in R [65]. Finally, the hierarchical tree was edited in the Interactive Tree Of Life (ITOL V6) online program [66].

#### Comparative analysis between historical and contemporary specimens.

Morphological data were analyzed from two groups of individuals grouped as historical specimens from museum collections and contemporary individuals captured in the wild. The dataset included measurements of six morphological traits, including head length, body length, tibia length, ear length, body weight, and forearm length. Sex was recorded as a categorical variable (i.e., Male, Female).

Analyses were conducted in R (version 4.4.2), using the packages readr, dplyr, tidyr, ggplot2, and ggpubr. Initially, sex was converted to a factor for group comparisons. Descriptive statistics (mean values) were calculated separately for males and females.

Welch’s two-sample t-tests were performed for each morphological trait comparing male and female individuals, accounting for unequal variances to assess sexual dimorphism. The results were organized by p-values to identify significant differences.

Furthermore, we compared morphological differences between historical (museum) and contemporary (wild) specimens. Two-sample t-tests were applied within each sex group to compare forearm lengths between historical and contemporary individuals.Data visualization included boxplots of morphological traits by sex, created with ggplot2. Faceted boxplots displayed trait distributions stratified by sex, while separate boxplots compared forearm lengths between historical and contemporary specimens.

Some data has been provided via this manuscript and the associated Supporting Information files. Other data can be found via Figshare at: https://figshare.com/articles/dataset/Morphological_data_i_Desmodus_rotundus_i_csv/27858219?file=51814880.

## Results

To assess sexual dimorphism in *Desmodus rotundus*, we performed two statistical analyses: one incorporating a series of morphological metrics and another focusing exclusively on forearm length. Welch’s t-tests indicated significant sexual dimorphism in forearm length (p = 6.8 × 10 ⁻ ⁵; FDR-adjusted p = 4.1 × 10 ⁻ ⁴; Bonferroni-adjusted p = 4.1 × 10 ⁻ ⁴) and tibia length (p = 0.003; FDR-adjusted p = 0.011; Bonferroni-adjusted p = 0.022). No other traits remained significant after correction for multiple testing.

A MANOVA on the six morphometric traits (body weight, head length, body length, tibia length, ear length, and forearm length) indicated a significant overall effect of sex (Pillai’s trace = 0.389, F(6,39)=4.14, p < 2.2 × 10 ⁻ ¹⁶). A few outliers (n = 12) were found in individuals from different localities. These outliers showed morphological differences from the bulk of samples and were retained in all analyses.

**Fig 3:**
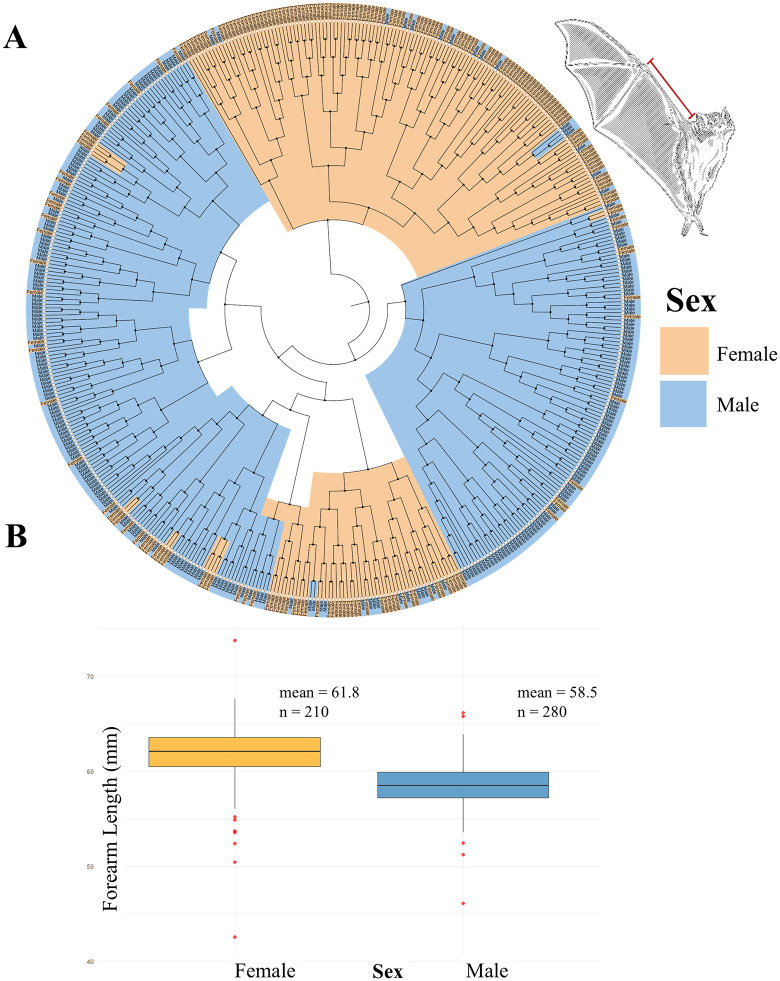
Comparison of forearm length in *Desmodus rotundus* by sex. **(A)** Distance tree by cluster analysis of *D. rotundus* according to forearm length from 490 historical and contemporary specimens. Orange: females. Blue: males. Note that the data tended to cluster in two groups based on sex. **(B)** Boxplot illustrating sexual dimorphism in forearm length. The median (central line) is higher in females than in males, indicating generally longer forearms in females. The 95% confidence interval for females spans 61.33–62.25 mm, and for males, 58.24–58.76 mm. The interquartile range (IQR) is slightly larger in females (3.05 mm) than in males (2.69 mm), suggesting greater variability within the female group. Outliers (red points) lie beyond the whiskers and represent individuals with forearm lengths notably outside the typical range for their sex. Mean forearm lengths were 61.8 mm for females and 58.5 mm for males.

Forearm length measurements (n = 490) revealed that females were larger than males (t = −12.677, df = 366.8, p < 2 × 10 ⁻ ¹⁶). Females (n = 210) exhibited a significantly greater mean forearm length (61.79 mm, SD = 3.37) compared to males (n = 280; m = 58.50 mm, SD = 2.21). The 95% confidence interval for females ranged from 61.33 to 62.25 mm, while for males it ranged from 58.24 to 58.76 mm. Interquartile ranges were 3.05 mm for females and 2.69 mm for males, suggesting slightly greater variability in female measurements. Furthermore, hierarchical clustering based on forearm length show a separation between males and females (Fig 3A and 3B).

We found a significant effect of sex on forearm length (t = −13.0, p < 2 × 10 ⁻ ¹⁶). The estimated mean forearm length for females was 61.8 mm while for males it was 58.5 mm, indicating that females have, on average, forearms 3.29 mm longer than males.

The model using sex as the only predictor explained 25.7% of the total variance in forearm length (R² = 0.26). The residual standard error was 2.77 mm, suggesting a moderate spread of individual values around the predicted means. These findings provide strong statistical support for sexual dimorphism in forearm length in this species, with females exhibiting significantly greater values than males.

**Fig 4:**
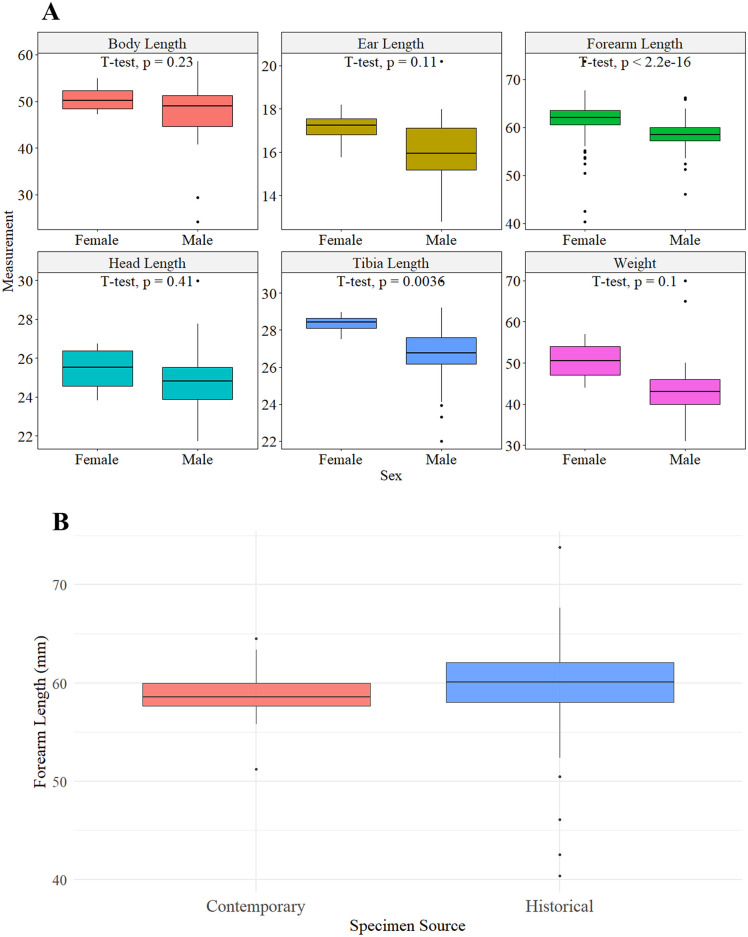
Morphometric comparisons by sex and origin in *Desmodus rotundus.* **(A)** Boxplots of six morphological traits (body length, ear length, forearm length, head length, tibia length, and body weight) comparing females and males. Each panel includes the result of a student’s t-test evaluating sexual dimorphism. Significant differences between sexes are observed in forearm and tibia length. **(B)** Comparison of forearm length between historical specimens and contemporary bats. Historical specimens tend to show a slightly broader range of variation. Forearm length was significantly greater in females than in males (t-test, p < 2 × 10 ⁻ ¹⁶), indicating a clear pattern of sexual dimorphism in this trait. Similarly, tibia length was greater in females, with a statistically significant difference (t-test, p = 0.0036). In contrast, no significant differences were observed between sexes in body length (p = 0.23), ear length (p = 0.11), head length (p = 0.41), or body weight (p = 0.1), although females tended to weigh more than males.

Comparisons between historical and contemporary specimens were conducted using student’s t-tests. Of the six measurements evaluated, only two showed statistically significant differences between sexes, forearm in historical specimens and tibia length, in contemporary specimens (Fig 4A).

Distribution of morphological traits by sex reveal sexual dimorphism based on forearm length of historical and contemporary specimens (Fig 4A and 4B).

There was no difference between historical (museum) and contemporary (wild-caught) specimens for females (t = −0.93, df = 208, p = 0.355), and males (t = −0.01, df = 278, p = 0.992).

## Discussion

Our study revealed female-biased sexual dimorphism in *Desmodus rotundus* in Colombia. Through both multivariate and univariate analyses, we found that *D. rotundus* females exhibit significantly larger forearm and tibia lengths compared to males. These findings align with prior reports of female-biased dimorphism in *D. rotundus* [30,31,67] and add to the growing body of literature on sex-based morphological variation in bats.

As an exploratory approach, we performed a principal components analysis and hierarchical clustering on a subset of 46 live-captured *D. rotundus* individuals with full morphometric data (see S1 Fig). Given the limited sample size and strong sex imbalance (only 4 females), results are not presented in the main text but may offer preliminary insights into multivariate morphological variation.

The second analysis, focused solely on forearm length in a larger sample of 490 individuals, reinforced these patterns with greater statistical power. Females had significantly longer forearms than males, with non-overlapping confidence intervals and consistent results across t-tests, linear regression, and clustering methods (Fig 3). The linear model explained over 25% of the variance in forearm length with sex as the sole predictor, a considerable proportion for a single biological variable, underscoring the strength of this dimorphic pattern in bats, as suggested by other authors [68–70].

In addition to multivariate methods, we performed univariate comparisons using student’s t-tests across six morphometric traits, including body length, ear length, forearm length, head length, tibia length, and weight, combining historic museum specimens and contemporary wild-caught animals (Fig 4). Significant sexual dimorphism was observed in traits forearm length and tibia length, with females being larger in both traits. No significant sex differences were found in other body metrics (i.e., body length, ear length, head length, and weight), although females tended to weigh more than males (Fig 4A). These findings further support a consistent pattern of dimorphism centered on locomotor traits rather than general body size, as observed by other authors [71,72].

Female-biased dimorphism in bats has been widely attributed to reproductive demands such as pregnancy, lactation, and pup rearing, which place substantial energetic and physiological burdens on females [73,74]. Larger body size in females may facilitate energy storage, thermoregulation, and load-carrying capacity during flight [73,74]. Our findings support this hypothesis and suggest that selective pressures, likely driven by ecological habits, are acting on *D. rotundus*, potentially leading to regional variation in forearm length, as has been observed in other Neotropical bat species [70–72]. This regional variation in forearm length should also be interpreted in the context of environmental and ecological variability. *D. rotundus* inhabits a wide range of environments—from tropical lowlands to montane regions—where factors such as prey availability, temperature, seasonal rainfall, and roosting conditions may impose distinct selective pressures that shape local morphology [75–77].

The presence of morphological outliers across several localities highlights the extent of individual variation within *D. rotundus* and likely reflects a combination of biological and ecological factors [78,79]. In total, we identified 12 outlier specimens—7 males and 5 females—distributed across 11 different localities. These individuals exhibited forearm lengths that fell beyond the expected range (based on the 1.5 × IQR rule) for their respective sex and locality groupings.

The most extreme outlier was a female from Agua de Dios (Cundinamarca) with a forearm length of 73.79 mm, more than 10 mm above the upper bound for females. This specimen, along with another unusually large female from Medina, Cundinamarca (67.65 mm), suggests the possibility of localized phenotypic divergence. These sites may exert selective pressures that promote larger body size, potentially in response to ecological strategy such as thermoregulatory demands or prey availability, as suggested by other authors [80–83]. Similarly, male outliers with notably small forearms, such as those from Chaparral (51.24 mm) and Venadillo Tolima (53.86 mm), and Tocaima, Cundinamarca (52.44 mm), also raise the possibility of ontogenetic or nutritional influences as evidenced in other bat species [84–86].

Given the wide ecological heterogeneity across Colombian landscapes, including transitional zones between lowland and montane habitats, there are unique opportunities to capture ecogeographic structure within populations [71,72,87,88]. The observed patterns of phenotypic, genetic, and environmental allometry differ markedly; notably, only environmental allometry reflects ontogenetic growth trends. This suggests that phenotypic allometry alone may not reliably predict how size and shape evolve over time [89]. For this reason, we chose to retain outliers in our analysis to preserve the inherent ecological variation present in natural populations. The geographic distribution and directionality of the observed relationships further underscore the importance of considering both environmental and ontogenetic factors when interpreting morphometric datasets, retaining the inherent ecological variation in natural populations [89–91].

The inclusion of historical museum specimens spanning over a century provided a valuable opportunity to assess long-term trends in morphological variation, a strategy widely used in evolutionary and ecological research [92–94]. While such collections offer valuable temporal breadth, they also present inherent limitations, including incomplete metadata, uncertain reproductive status, and unknown age of individuals [95]. Despite these constraints, the consistent pattern of female-biased sexual dimorphism across regions supports the interpretation of a biologically meaningful signal. This finding is in line with other studies demonstrating the utility of museum collections for detecting macroecological trends, even in the presence of data gaps [96,97].

Discrepancies in the literature regarding sexual dimorphism in *D. rotundus* may stem from regional variation, small or unbalanced sample sizes, and lack of control for confounding variables [98,99].

Beyond evolutionary implications, our findings may hold relevance for understanding behavior and disease ecology. Morphological differences between sexes can influence activity patterns, dispersal, and social structure [11, 100], all of which are relevant to the transmission dynamics of the rabies virus and other zoonoses in vampire bats [101].

### Limitations

The reliance on measurements from the right forearm precluded assessment of bilateral asymmetry, an important indicator of developmental stability. For example, fluctuating asymmetry (FA), particularly in forelimbs, has been reported in bats [102]. Studies in *Saccopteryx bilineata* and *Carollia perspicillata* have been associated FA with reproductive success and fitness component [103,104]. Similarly, the use of historical specimens introduced potential biases, including uneven geographic representation, unknown age or reproductive condition and potential variation of size during preservation [105]. Lastly, additional traits—such as wingspan, digit length, wing loading, cranial metrics, and glands and non-glandular odour-producing structures —were not available due to the state of conservation of most of the specimens, and it could provide a more comprehensive view of functional dimorphism [106,107].

Outlier specimens that fall within the size range typical of the opposite sex may reflect individual variation due to factors such as age, health condition, or environmental stress [43,44]. In some cases, measurement errors or incomplete sexual dimorphism could also account for the overlap between groups [45,46]. Future studies using digital imaging and geometric morphometrics may offer improved precision and new insights into shape-based dimorphism [108].

## Conclusion

Our morphometric analyses reveal modest but consistent female-biased sexual dimorphism in *Desmodus rotundus*, particularly in forearm and tibia length. This pattern likely reflects a combination of reproductive, ecological, and behavioral pressures acting on females. The inclusion of both historical and contemporary specimens strengthens the reliability of our findings, while also highlighting the value and limitations of museum-based research. Future studies incorporating genetic and ecological variables will be essential to fully understand the evolutionary drivers of sexual dimorphism in this species. As anthropogenic pressures continue to alter bat habitats and behaviors, such knowledge will be crucial for species conservation and for mitigating zoonotic disease risks.

## Supporting information

S1 Table Morphometric measurements of *Desmodus rotundus* specimens. Morphometric data collected from *Desmodus rotundus* specimens, including forearm length and other standard body measurements.
(CSV)

S2 Table Principal Component Analysis (PCA) of six morphometric traits in *Desmodus rotundus.* This table summarizes the results of a Principal Component Analysis conducted on six morphological traits. The upper section shows the standard deviation, proportion of variance, and cumulative proportion of variance explained by the first six principal components (PC1–PC6). The lower section lists the loadings of each morphological trait on the corresponding principal components, indicating the contribution of each variable to the respective PC.
(CSV)

S1 Fig Principal Component Analysis (PCA) of morphometric data. Principal Component Analysis (PCA) plot based on the morphometric measurements of *Desmodus rotundus* (n = 46).
(TIF)

S1 Text R scripts used for statistical analyses and plots. This file contains the R code used for data processing, statistical analyses, and generation of figures presented in the manuscript.
(DOCX)

## References

[1] Scheffer KC, Barros RF, Iamamoto K, Mori E, Asano KM, Lima JYO, et al. *Desmodus rotundus* - Biologia y comportamiento [Ph.D. Thesis]. Instituto Pasteur; 2022. doi: 10.37885/220308371

[2] Sanchez-Gomez WS, Selem-Salas CI, Cordova-Aldana DI, Erales-Villamil JA. Common vampire bat (*Desmodus rotundus*) abundance and frequency of attacks to cattle in landscapes of Yucatan, Mexico. Trop Anim Health Prod. 2022;54(2):130. doi: 10.1007/s11250-022-03122-w 35258761

[3] Brown N, Escobar LE. A review of the diet of the common vampire bat (*Desmodus rotundus*) in the context of anthropogenic change. Mamm Biol. 2023;:1–21. doi: 10.1007/s42991-023-00358-3 37363038 PMC10258787

[4] Wilson DE, Wilson DE, Mittermeier RA. Handbook of the mammals of the world: hoofed mammals. 2nd ed. Barcelona: Lynx Edicions; 2011.

[5] Iturra-Herrera L, Brito-Carrasco B, Daigre M, Arce P, Arriagada-Gajewski M. Ampliación del rango de distribución sur de Desmodus rotundus (É. Geoffroy Saint Hilaire, 1810), Islote de Pupuya. Región del Libertador Bernardo O´Higgins, Chile Central. Bol Mus Nac Hist Nat. 2020;68(2019):5–12. Available from: https://publicaciones.mnhn.gob.cl/668/w3-article-95862.html

[6] Van de Vuurst P, Díaz MM, Rodríguez-San Pedro A, Allendes JL, Brown N, Gutiérrez JD, et al. A database of common vampire bat reports. Sci Data. 2022;9(1):57. doi: 10.1038/s41597-022-01140-9 35173163 PMC8850563

[7] Gardner AL. Mammals of South America, 1st ed: marsupials, xenarthrans, shrews, and bats. University of Chicago Press; 2019.

[8] Greenhall AM, Joermann G, Schmidt U, Seidel MR. Desmodus rotundus. Mamm Species. 1983;(202):1–6. doi: 10.2307/3503895

[9] Arellano-Sota C. Biology, ecology, and control of the vampire bat. Rev Infect Dis. 1988;10 Suppl 4:S615-9. doi: 10.1093/clinids/10.supplement_4.s615 3060955

[10] Bolívar-Cimé B, Flores-Peredo R, García-Ortíz SA, Murrieta-Galindo R, Laborde J. Influence of landscape structure on the abundance of *Desmodus rotundus* (Geoffroy 1810) in northeastern Yucatan, Mexico. Ecosist Recur Agropec. 2019;6(17):263–71. doi: 10.19136/era.a6n17.1968

[11] Wilkinson GS. The social organization of the common vampire bat. Behav Ecol Sociobiol. 1985;17:111–21. doi: 10.1007/BF00299243

[12] Lord RD. Seasonal reproduction of vampire bats and its relation to seasonality of bovine rabies. J Wildl Dis. 1992;28(2):292–4. doi: 10.7589/0090-3558-28.2.292 1602584

[13] Souza ACF, Santos FC, Bastos DSS, Sertorio MN, Teixeira JPG, Fernandes KM, et al. Reproductive functions in *Desmodus rotundus*: a comparison between seasons in a morphological context. PLoS One. 2018;13(10):e0205023. doi: 10.1371/journal.pone.0205023 30332444 PMC6192620

[14] Delpietro HA, Russo RG, Carter GG, Lord RD, Delpietro GL. Reproductive seasonality, sex ratio and philopatry in Argentina’s common vampire bats. R Soc Open Sci. 2017;4(4):160959. doi: 10.1098/rsos.160959 28484615 PMC5414252

[15] de Mello VVC, Placa AJV, Lee DAB, Franco EO, Lima L, Teixeira MMG, et al. Molecular detection of blood-borne agents in vampire bats from Brazil, with the first molecular evidence of *Neorickettsia* sp. in *Desmodus rotundus* and *Diphylla ecaudata*. Acta Trop. 2023;244:106945. doi: 10.1016/j.actatropica.2023.106945 37207993

[16] Hughes W. Maladies of the modern vampire. In: Bacon S, editor. The palgrave handbook of the vampire. Cham: Springer International Publishing; 2023. p. 1–23.

[17] Alves RS, do Canto Olegário J, Weber MN, da Silva MS, Canova R, Sauthier JT, et al. Detection of coronavirus in vampire bats (*Desmodus rotundus*) in southern Brazil. Transbound Emerg Dis. 2022;69(4):2384–9. doi: 10.1111/tbed.14150 33977671 PMC8242716

[18] Rocha F, Dias RA. The common vampire bat *Desmodus rotundus* (Chiroptera: Phyllostomidae) and the transmission of the rabies virus to livestock: a contact network approach and recommendations for surveillance and control. Prev Vet Med. 2020;174:104809. doi: 10.1016/j.prevetmed.2019.104809 31756671

[19] Gomes M. Importância médica dos morcegos vampiros (*Desmodus rotundus*) naecologia de doenças e na pesquisa biomédica. Revista Tópicos. 2024;2:15. doi: 10.5281/zenodo.14201994

[20] Heestermans M, Poenou G, Hamzeh-Cognasse H, Cognasse F, Bertoletti L. Anticoagulants: a short history, their mechanism of action, pharmacology, and indications. Cells. 2022;11(20):3214. doi: 10.3390/cells11203214 36291080 PMC9600347

[21] Cohen O, Santagata D, Ageno W. Novel horizons in anticoagulation: the emerging role of factor XI inhibitors across different settings. Haematologica. 2024;109(10):3110–24. doi: 10.3324/haematol.2023.283682 38779744 PMC11443408

[22] Espinoza-Gómez A, Moreno-Santillán DD, Juárez-Maldonado R, Gutiérrez EG, Salazar MaI, Alonso-Palomares LA, et al. Identification of viral RNA sequences in vampire bats (*Desmodus rotundus*) from central Mexico. RevMexBiodiv. 2022;93:e934021. doi: 10.22201/ib.20078706e.2022.93.4021

[23] Cárdenas-Canales EM, Stockmaier S, Cronin E, Rocke TE, Osorio JE, Carter GG. Social effects of rabies infection in male vampire bats (*Desmodus rotundus*). Biol Lett. 2022;18(9):20220298. doi: 10.1098/rsbl.2022.0298 36069068 PMC9449815

[24] Albuquerque NK, Silva SP, Aragão CF, Cunha TCAS, Paiva FAS, Coelho TFSB, et al. Virome analysis of *Desmodus rotundus* tissue samples from the Amazon region. BMC Genom. 2024;25(1):34. doi: 10.1186/s12864-023-09950-w 38177994 PMC10768307

[25] Kunz TH, Braun de Torrez E, Bauer D, Lobova T, Fleming TH. Ecosystem services provided by bats. Ann N Y Acad Sci. 2011;1223:1–38. doi: 10.1111/j.1749-6632.2011.06004.x 21449963

[26] Chambers AC. Virological vampires. In: Bacon S, editor. The palgrave handbook of the vampire. Cham: Springer International Publishing; 2023. p. 1–20.

[27] Hudson D. Vampires and the anthropocene. In: Bacon S, editor. The palgrave handbook of the vampire. Cham: Springer International Publishing; 2024. p. 1729–49.

[28] Carter GG, Farine DR, Crisp RJ, Vrtilek JK, Ripperger SP, Page RA. Development of new food-sharing relationships in vampire bats. Curr Biol. 2020;30(7):1275-1279.e3. doi: 10.1016/j.cub.2020.01.055 32197089

[29] Crisp RJ, Brent LJN, Carter GG. Social dominance and cooperation in female vampire bats. R Soc Open Sci. 2021;8(7):210266. doi: 10.1098/rsos.210266 34295524 PMC8261227

[30] Mann CS, Aulagnier S. Biométrie crânienne et brachiale de *Desmodus rotundus* (Chiroptera, Phyllostomidae) de Guyane Française. Mammalia. 1993;57(4):589–600. doi: 10.1515/mamm.1993.57.4.589

[31] Ralls K. Mammals in which females are larger than males. Q Rev Biol. 1976;51(2):245–76. doi: 10.1086/409310 785524

[32] Orihuela J. Skull variation of the vampire bat *Desmodus rotundus* (Chiroptera: Phyllostomidae): taxonomic implications for the Cuban fossil vampire bat *Desmodus puntajudensis*. Chiropt Neotrop. 2011;17(1):863–76.

[33] Delpietro HA, Russo RG. Observations of the common vampire bat (*Desmodus rotundus*) and the hairy-legged vampire bat (*Diphylla ecaudata*) in captivity. Mamm Biol. 2002;67(2):65–78. doi: 10.1078/1616-5047-00011

[34] Adams DM, Nicolay C, Wilkinson GS. Patterns of sexual dimorphism and mating systems. In: Fleming TH, Dávalos LM, Mello MAR, editors. Phyllostomid bats: a unique mammalian radiation. University of Chicago Press; 2020. doi: 10.7208/chicago/9780226696263.003.0013

[35] Seetahal JFR, Streicker DG, Beerli P, Sahadeo N, Lemey P, Sanchez-Vazquez MJ, et al. Population genetics, phylogeography and gene flow of mainland and island vampire bat (*Desmodus rotundus*) populations: an investigation into mainland-island bat movement. bioRxiv. 2024:2024-01. doi: 10.1101/2024.01.29.577751

[36] QGIS Development Team. QGIS Geographic Information System. Open Source Geospatial Foundation Project; 2019. Available from: http://qgis.osgeo.org

[37] National Research Council (US) Institute for Laboratory Animal Research. The development of science-based guidelines for laboratory animal care: Proceedings of the November 2003 international workshop. Washington: National Academies Press; 2004. 20669462

[38] Sikes RS, Animal Care and Use Committee of the American Society of Mammalogists. 2016 Guidelines of the American Society of Mammalogists for the use of wild mammals in research and education. J Mammal. 2016;97(3):663–88. doi: 10.1093/jmammal/gyw078 29692469 PMC5909806

[39] Diven K. Inhalation anesthetics in rodents. Lab Anim (NY). 2003;32(3):44–7. doi: 10.1038/laban0303-44 12601389

[40] Pérez-Torres J, Herrera-Sepúlveda MT, Pantoja-Peña G. A new device for capturing social bats in caves. Mastozool Neotrop. 2020;27:206–10. doi: 10.31687/saremMN.20.27.1.0.09

[41] Chaves-Ramírez S, Castillo-Salazar C, Sánchez-Chavarría M, Solís-Hernández H, Chaverri G. Comparing the efficiency of monofilament and regular nets for capturing bats. R Soc Open Sci. 2021;8(12):211404. doi: 10.1098/rsos.211404 34909218 PMC8652279

[42] Kunz TH, Fenton MB. Bat ecology. Chicago: University of Chicago Press; 2005.

[43] Weterings R, Umponstira C. Bodyweight-forearm ratio, cranial morphology and call frequency relate to prey selection in insectivorous bats. Electronic J Biol. 2014;10:21–7.

[44] Findley JS, Wilson DE. Ecological significance of chiropteran morphology. In: Kunz TH, editor. Ecology of bats. Boston: Springer; 1982. p. 243–60.

[45] Sánchez MS, Carrizo LV. Forelimb bone morphology and its association with foraging ecology in four families of neotropical bats. J Mammal Evol. 2020;28(1):99–110. doi: 10.1007/s10914-020-09526-5

[46] Evolutionary history of bats: fossils, molecules and morphology. J Mammal. 2013;94:520–1. doi: 10.1644/12-MAMM-R-305

[47] Díaz MM, Solari S, Gregorin R, Aguirre LF, Barquez RM. Clave de identificación de los murciélagos neotropicales. San Miguel de Tucumán: Programa de conservación de los murciélagos de Argentina; 2021.

[48] Lopez-Baucells A, Rocha R, Bernard E, Palmeirim J, Meyer C. Field guide to amazonian bats. 1st ed. Manaos: INPA; 2016.

[49] Castillo-Figueroa D. Does Bergmann’s rule apply in bats? Evidence from two neotropical species. Neotrop Biodivers. 2022;8(1):200–21. doi: 10.1080/23766808.2022.2075530

[50] Dietz C, Dietz I, Siemers BM. Wing measurement variations in the five european horseshoe bat species (Chiroptera: Rhinolophidae). J Mammal. 2006;87(6):1241–51. doi: 10.1644/05-mamm-a-299r2.1

[51] Lisón F, Haz Á, González‐Revelles C, Calvo JF. Sexual size dimorphism in greater mouse‐eared bat *Myotis myotis* (Chiroptera: Vespertilionidae) from a Mediterranean region. Acta Zool. 2012;95(2):137–43. doi: 10.1111/azo.12012

[52] Cruz Rodríguez CA, Cárdenas JS. Colección de Mastozoología del Museo de La Salle – Bogotá (MLS). MaNo. 2020;6(1):mn0111. doi: 10.47603/manovol6n1.mn0111

[53] García-R S. Colección de Mamíferos, Museo de Historia Natural C.J. Marinkelle, Universidad de los Andes (ANDES-M), Colombia. MaNo. 2020;6(1):mn0109. doi: 10.47603/manovol6n1.mn0109

[54] López-Arévalo HF, Montenegro OL, Cárdenas-González C. Colección de mamíferos “Alberto Cadena García” Instituto de Ciencias Naturales, Universidad Nacional de Colombia (ICN). MaNo. 2020;6:199–199. doi: 10.47603/mano.v6n2.199

[55] Lozano-Flórez J, Cifuentes-Acevedo S, Borja-Acosta KG, Gómez-Posada C. Colección de Mamíferos del Instituto Humboldt (IAvH-M). MaNo. 2020;6(1):mn0122. doi: 10.47603/manovol6n1.mn0122

[56] Zurc D, Bustamante A, Arbeláez Rendón E, Alzate-Zapata OS. Colección de Mamíferos, Museo de Ciencias Naturales de La Salle del Instituto Tecnológico Metropolitano (CSJ-m). MaNo. 2020;6(1):mn0110. doi: 10.47603/manovol6n1.mn0110

[57] Moreno G, Yantén A, Patiño Quíroz MF, Ramírez D, Hernández OF, Sánchez F. Colección Mastozoológica, Museo de Historia Natural UNILLANOS MHNU-M. MaNo. 2020;6(2):175. doi: 10.47603/mano.v6n2.175

[58] Ramírez-Chaves HE, Velasquez-Guarín D, Mejía-Fontecha IY, Ocampo-Velásquez JD, Castaño Ramírez ND. Colección de mamíferos (Mammalia) del Museo de Historia Natural de la Universidad de Caldas, Colombia. Biota Colomb. 2020;21(2):156–66. doi: 10.21068/c2020.v21n02a11

[59] Rodriguez-Bolaños A. Colección de Mamíferos, Museo de Historia Natural de la Universidad Distrital Francisco José de Caldas (MHNUD-M). MaNo. 2020;6(1):mn0120. doi: 10.47603/manovol6n1.mn0120

[60] R: the R project for statistical computing. [cited 2025 May 18]. Available from: https://www.r-project.org/

[61] Welch BL. The generalisation of student’s problems when several different population variances are involved. Biometrika. 1947;34(1–2):28–35. doi: 10.1093/biomet/34.1-2.28 20287819

[62] Benjamini Y, Hochberg Y. Controlling the false discovery rate: a practical and powerful approach to multiple testing. J R Stat Soc Ser B Methodol. 1995;57:289–300. doi: 10.1111/j.2517-6161.1995.tb02031.x

[63] Rencher AC. A review of “methods of multivariate analysis, second edition”. IIE Trans. 2005;37(11):1083–5. doi: 10.1080/07408170500232784

[64] Galili T. dendextend: an R package for visualizing, adjusting and comparing trees of hierarchical clustering. Bioinformatics. 2015;31(22):3718–20. doi: 10.1093/bioinformatics/btv428 26209431 PMC4817050

[65] Paradis E, Claude J, Strimmer K. APE: analyses of phylogenetics and evolution in R language. Bioinformatics. 2004;20(2):289–90. doi: 10.1093/bioinformatics/btg412 14734327

[66] Letunic I, Bork P. Interactive Tree Of Life (iTOL) v5: an online tool for phylogenetic tree display and annotation. Nucleic Acids Res. 2021;49(W1):W293–6. doi: 10.1093/nar/gkab301 33885785 PMC8265157

[67] Gomes MN, Uieda W. Abrigos diurnos, composição de colônias, dimorfismo sexual e reprodução do morcego hematófago *Desmodus rotundus* (E. Geoffroy) (Chiroptera, Phyllostomidae) no Estado de São Paulo, Brasil. Rev Bras Zool. 2004;21(3):629–38. doi: 10.1590/s0101-81752004000300025

[68] Castillo-Figueroa D. Sexual size dimorphism in 28 Neotropical bat species fails to obey Rensch’s rule. Acta Chiropt. 2024;25(2):311–21. doi: 10.3161/15081109acc2023.25.2.010

[69] Wu H, Jiang T, Huang X, Feng J. Patterns of sexual size dimorphism in horseshoe bats: testing Rensch’s rule and potential causes. Sci Rep. 2018;8(1):2616. doi: 10.1038/s41598-018-21077-7 29422495 PMC5805768

[70] Orkney A, Boerma DB, Hedrick BP. Evolutionary integration of forelimb and hindlimb proportions within the bat wing membrane inhibits ecological adaptation. Nat Ecol Evol. 2024;9(1):111–23. doi: 10.1038/s41559-024-02572-9 39487310

[71] Reyes-Amaya N, Jerez A, Flores D. Morphology and postnatal development of lower hindlimbs in *Desmodus rotundus* (Chiroptera: Phyllostomidae): a comparative study. Anat Rec (Hoboken). 2017;300(12):2150–65. doi: 10.1002/ar.23646 28805956

[72] Castillo-Figueroa D. Ecological morphology of neotropical bat wing structures. Zool Stud. 2020;59:e60. doi: 10.6620/ZS.2020.59-60 34140977 PMC8181164

[73] McGuire LP, Boyles JG. Energetics of foraging bats. In: Russo D, Fenton B, editors. A natural history of bat foraging. Academic Press; 2024. p. 173–98.

[74] Maucieri DG, Ashbaugh AJ, Theodor JM. Sexual dimorphism in bat wing morphology — variation among foraging styles. Can J Zool. 2021;99: 953–60. doi: 10.1139/cjz-2021-0035

[75] Adams RA, Hayes MA. Assemblage-level analysis of sex-ratios in Coloradan bats in relation to climate variables: a model for future expectations. Glob Ecol Conserv. 2018;14:e00379. doi: 10.1016/j.gecco.2018.e00379

[76] Barclay RMR. Variable variation: annual and seasonal changes in offspring sex ratio in a bat. PLoS One. 2012;7(5):e36344. doi: 10.1371/journal.pone.0036344 22570704 PMC3343075

[77] Culina A, Linton DM, Macdonald DW. Age, sex, and climate factors show different effects on survival of three different bat species in a woodland bat community. Glob Ecol Conserv. 2017;12:263–71. doi: 10.1016/j.gecco.2017.11.009

[78] Ayala-Berdon J, Orozco-Lugo L, Medina-Bello KI. Coping with seasons: morphological and physiological adjustments along the year in vampire bats (*Desmodus rotundus*) from central Mexico. Res Sq [Preprint]. 2025 [cited 2025 Jun 12]. Available from: doi: 10.21203/rs.3.rs-5800286/v

[79] Wasserman D, Nash DJ. Variation in body size, hair length, and hair density in the deer mouse *Peromyscus maniculatus* along an altitudinal gradient. Ecography. 1979;2(2):115–8. doi: 10.1111/j.1600-0587.1979.tb00689.x

[80] Ulian CMV, Rossi MN. Intraspecific variation in body size and sexual size dimorphism, and a test of Rensch’s rule in bats. Acta Zool. 2017;98(4):377–86. doi: 10.1111/azo.12183

[81] Santana SE, Cheung E. Go big or go fish: morphological specializations in carnivorous bats. Proc Biol Sci. 2016;283(1830):20160615. doi: 10.1098/rspb.2016.0615 27170718 PMC4874722

[82] Arbour JH, Curtis AA, Santana SE. Sensory adaptations reshaped intrinsic factors underlying morphological diversification in bats. BMC Biol. 2021;19(1):88. doi: 10.1186/s12915-021-01022-3 33931060 PMC8086122

[83] Lomolino MV. Body size evolution in insular vertebrates: generality of the island rule. J Biogeogr. 2005;32:1683–99. doi: 10.1111/j.1365-2699.2005.01314.x

[84] Fleming TH. The relationship between body size, diet, and habitat use in frugivorous bats, genus *Carollia* (Phyllostomidae). J Mammal. 1991;72:493–501. doi: 10.2307/1382132

[85] Brigham RM. Prey detection, dietary niche breadth, and body size in bats: why are aerial insectivorous bats so small? Am Nat. 1991;137(5):693–703. doi: 10.1086/285188

[86] Davy CM, von Zuben V, Kukka PM, Gerber BD, Slough BG, Jung TS. Rapidly declining body size in an insectivorous bat is associated with increased precipitation and decreased survival. Ecol Appl. 2022;32(7):e2639. doi: 10.1002/eap.2639 35443093 PMC10078423

[87] Lintott PR, Mathews F. Basic mathematical errors may make ecological assessments unreliable. Biodivers Conserv. 2018;27(1):265–7. doi: 10.1007/s10531-017-1418-5 31997854 PMC6956893

[88] Benhadi-Marín J. A conceptual framework to deal with outliers in ecology. Biodivers Conserv. 2018;27(12):3295–300. doi: 10.1007/s10531-018-1602-2

[89] Cheverud JM. Relationships among ontogenetic, static, and evolutionary allometry. Am J Phys Anthropol. 1982;59(2):139–49. doi: 10.1002/ajpa.1330590204 7149015

[90] Bischof EA, Schlüter N, Lehmann J. Geometric morphometric analysis of morphologic disparity, intraspecific variation and ontogenetic allometry of beyrichitine ammonoids. PLoS One. 2022;17(2):e0263524. doi: 10.1371/journal.pone.0263524 35143539 PMC8830730

[91] Mandarim-de-Lacerda CA. Ontogenetic and phylogenetic allometry (bivariate and multivariate) for young morphologists. Int J Morphol. 2019;37(2):466–72. doi: 10.4067/s0717-95022019000200466

[92] Bakker FT, Antonelli A, Clarke JA, Cook JA, Edwards SV, Ericson PGP, et al. The Global Museum: natural history collections and the future of evolutionary science and public education. PeerJ. 2020;8:e8225. doi: 10.7717/peerj.8225 32025365 PMC6993751

[93] Holmes MW, Hammond TT, Wogan GOU, Walsh RE, LaBarbera K, Wommack EA, et al. Natural history collections as windows on evolutionary processes. Mol Ecol. 2016;25(4):864–81. doi: 10.1111/mec.13529 26757135 PMC4755843

[94] Shultz AJ, Adams BJ, Bell KC, Ludt WB, Pauly GB, Vendetti JE. Natural history collections are critical resources for contemporary and future studies of urban evolution. Evol Appl. 2020;14(1):233–47. doi: 10.1111/eva.13045 33519967 PMC7819571

[95] Fearing A, Faulkner K, Smith P, Humbrecht E, Kyne PM, Feldheim KA, et al. Assessing confidence in zoological specimen collection metadata for use in scientific studies. J Nat Conserv. 2025;84:126815. doi: 10.1016/j.jnc.2024.126815

[96] Graham CH, Ferrier S, Huettman F, Moritz C, Peterson AT. New developments in museum-based informatics and applications in biodiversity analysis. Trends Ecol Evol. 2004;19(9):497–503. doi: 10.1016/j.tree.2004.07.006 16701313

[97] Ponder WF, Carter GA, Flemons P, Chapman RR. Evaluation of museum collection data for use in biodiversity assessment. Conserv Biol. 2001;15(3):648–57. doi: 10.1046/j.1523-1739.2001.015003648.x

[98] Magory T, Kiat Y, Sharon H, Levin E. An alternative hypothesis for the evolution of sexual segregation in endotherms. Glob Ecol Biogeogr. 2021;30:2420–30. doi: 10.1111/geb.13393

[99] Paltrinieri L, Razgour O, Santini L, Russo D, Aihartza J, Aizpurua O, et al. The effects of climate on bat morphology across space and time. Ecography. 2025;2025(7):e07663. doi: 10.1002/ecog.07663

[100] Sánchez-Hernández C, Romero-Almaraz M de L, Wooten MC, Schnell GD, Kennedy ML. Speed in flight of common vampire bats (*Desmodus rotundus*). Southwest Nat. 2006;51(3):422–5. doi: 10.1894/0038-4909(2006)51[422:sifocv]2.0.co;2

[101] Rico-Chávez O, Flores-Pérez N, Martínez-Pérez KU, del Carmen Villalobos-Segura M, Ávila-Flores R. Bats, pathogen diversity and rabies in a changing Neotropic landscape. In: Acosta-Jamett G, Chaves A, editors. Ecology of wildlife diseases in the neotropics. Cham: Springer; 2024. p. 185–212.

[102] López-Aguirre C, Hand SJ, Koyabu D, Tu VT, Wilson LAB. Prenatal developmental trajectories of fluctuating asymmetry in bat humeri. Front Cell Dev Biol. 2021;9:639522. doi: 10.3389/fcell.2021.639522 34124034 PMC8187808

[103] Monteiro LR, Mellado B, Nogueira MR, de Morais-Jr MM. Individual asymmetry as a predictor of fitness in the bat *Carollia perspicillata*. J Evol Biol. 2019;32(11):1207–29. doi: 10.1111/jeb.13522 31420901

[104] Voigt CC, Heckel G, Mayer F. Sexual selection favours small and symmetric males in the polygynous greater sac-winged bat *Saccopteryx bilineata* (Emballonuridae, Chiroptera). Behav Ecol Sociobiol. 2005;57:457–64. doi: 10.1007/s00265-004-0874-6

[105] Meineke EK, Daru BH. Bias assessments to expand research harnessing biological collections. Trends Ecol Evol. 2021;36(12):1071–82. doi: 10.1016/j.tree.2021.08.003 34489117

[106] Conenna I, Santini L, Rocha R, Monadjem A, Cabeza M, Russo D. Global patterns of functional trait variation along aridity gradients in bats. Glob Ecol Biogeogr. 2021;30:1014–29. doi: 10.1111/geb.13278

[107] Muñoz‐Romo M, Page RA, Kunz TH. Redefining the study of sexual dimorphism in bats: following the odour trail. Mammal Rev. 2021;51(2):155–77. doi: 10.1111/mam.12232

[108] Merckx J, Van Roie M, Gómez-Zurita J, Dekoninck W. From theory to practice: a photographic inventory of museum collections to optimize collection management. Biodiv Inf. 2018;13. doi: 10.17161/bi.v13i0.7036

