# Supplementary figures and images for: Assessing sexual dimorphism in the common vampire bat, Desmodus rotundus
Source: PLoS One. 2026 Jan 21;21(1):e0320169. doi: 10.1371/journal.pone.0320169 (PMC12822921; doi:10.1371/journal.pone.0320169)

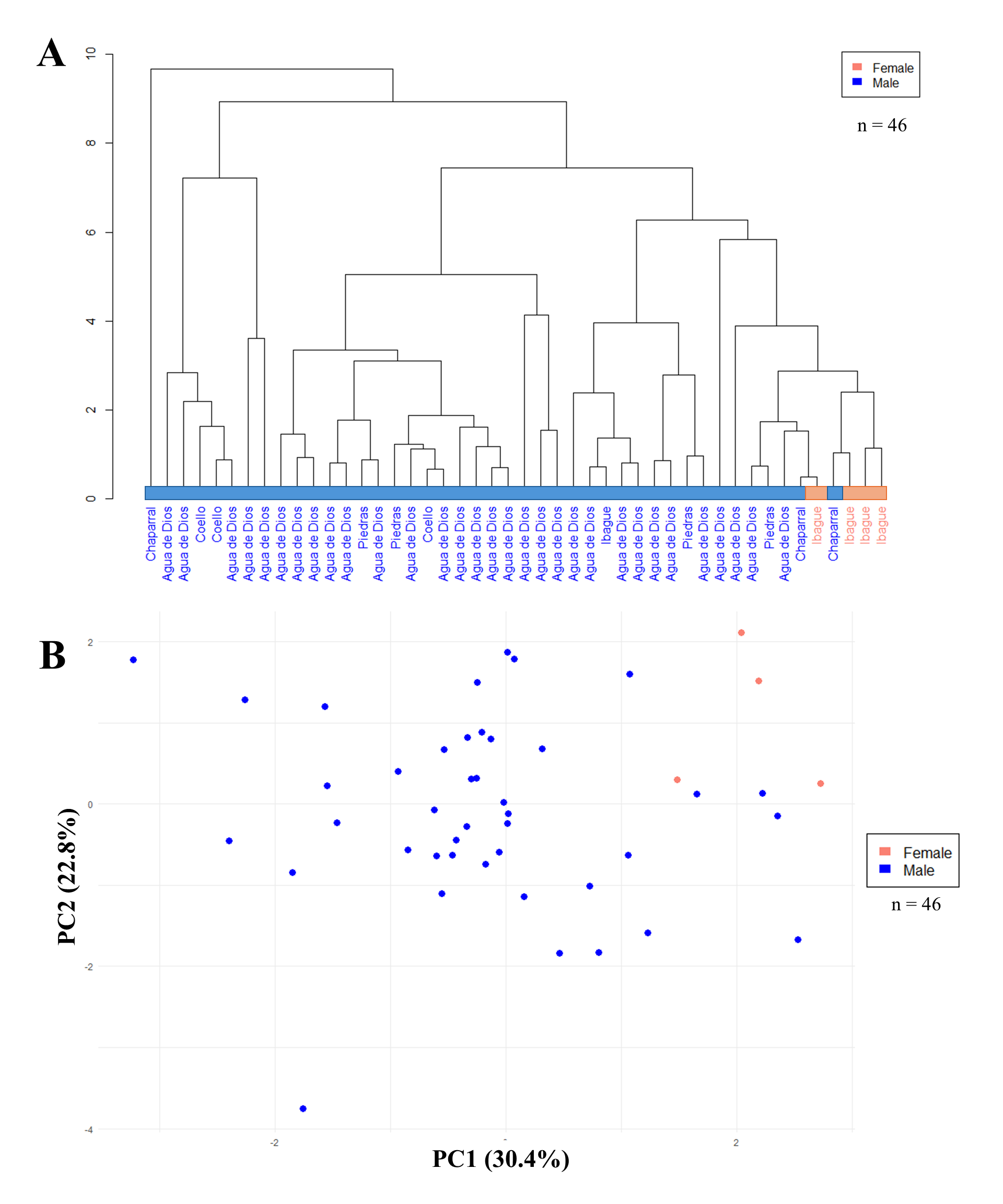

Supplement: S1 Fig — Principal Component Analysis (PCA) plot based on the morphometric measurements of Desmodus rotundus (n = 46). (TIF) [file pone.0320169.s003.tif]
