# Supplementary material for: Assessing sexual dimorphism in the common vampire bat, Desmodus rotundus
Source: PLoS One. 2026 Jan 21;21(1):e0320169. doi: 10.1371/journal.pone.0320169 (PMC12822921; doi:10.1371/journal.pone.0320169)
Supplement: S1 Text — This file contains the R code used for data processing, statistical analyses, and generation of figures presented in the manuscript. (DOCX) [file pone.0320169.s004.docx]

**Full code used for all analyses and visualization**

- **Summary stats**

library(ggplot2)

library(dplyr)

library(readr)

# Load the dataset

file_path <- "morphological_data.csv"

data <- read_csv(file_path)

# Inspect the data

str(data)

summary(data)

# Remove rows with missing values for key variables

clean_data <- data %>%

filter(!is.na(forearm), !is.na(sex))

# Group by sex and statistics

summary_stats <- clean_data %>%

group_by(sex) %>%

summarise(

n = n(),

mean = mean(forearm),

sd = sd(forearm),

IQR = IQR(forearm),

lower_CI = mean - qt(0.975, df = n - 1) * sd / sqrt(n),

upper_CI = mean + qt(0.975, df = n - 1) * sd / sqrt(n)

)

#Print result

print(summary_stats)

- **Regression model**

library(ggplot2)

library(dplyr)

library(readr)

# Inspect the data

str(data)

summary(data)

# Remove rows with missing values for key variables

clean_data <- data %>%

filter(!is.na(forearm), !is.na(sex))

# Convert sex to a factor

clean_data$sex <- as.factor(clean_data$sex)

ggplot(clean_data, aes(x = sex, y = forearm, fill = sex)) +

geom_boxplot(alpha = 0.7) +

scale_fill_manual(values = c("Male" = "#1f78b4", "Female" = "orange")) +

labs(title = "Forearm Length by Sex", y = "forearm") +

theme_minimal()

# Linear regression model

model <- lm(forearm ~ sex, data = clean_data)

summary(model)

- **t-student test**

# Delete row with missing data in both groups

male_data <- male_data[complete.cases(male_data$forearm), ]

female_data <- female_data[complete.cases(female_data$forearm), ]

# Verify numeric data without missing data

if(all(sapply(male_data$forearm, is.numeric)) && all(sapply(female_data$forearm, is.numeric))) {

# t-Student test

t_test_result <- t.test(male_data$forearm, female_data$forearm)

# Print result

print(t_test_result)

- **t-student test comparing historical samples with contemporary samples**

library(readr)

library(dplyr)

library(tidyr)

library(ggplot2)

library(ggpubr)

# Read data

file_path <- "C:/Users/analorena/Desktop/DIMORPHISM PAPER/PLOS ONE SUBMISSION/S1 Table.csv"

data <- read_csv(file_path)

class(data)

# Convert sex as a factor

data$sex <- as.factor(data$sex)

# Compare

morpho_vars <- c("head", "body", "tibia", "ear", "weight", "forearm")

results <- data.frame(Variable = character(),

Mean_Male = numeric(),

Mean_Female = numeric(),

p_value = numeric(),

stringsAsFactors = FALSE)

# t-test

for (var in morpho_vars) {

male_values <- data %>% filter(sex == "Male") %>% pull(var)

female_values <- data %>% filter(sex == "Female") %>% pull(var)

test <- t.test(male_values, female_values, var.equal = FALSE)

results <- rbind(results, data.frame(

Variable = var,

Mean_Male = mean(male_values, na.rm = TRUE),

Mean_Female = mean(female_values, na.rm = TRUE),

p_value = test$p.value

))

}

data_filtered <- data %>%

dplyr::select(5:11)

unique(data_filtered$sex)

t.test(body~sex, data = data_filtered , var.equal = TRUE)

names(data_filtered) <- c( "Head Length" , "Body Length" , "Tibia Length" , "Ear Length" , "Weight" , "Forearm Length", "Sex" )

gather_data <- gather(data_filtered,

key = "traits",

value = "Measurement",

-Sex)

gather_data

ggplot(gather_data, aes(sex, value, fill = traits)) +

geom_boxplot() +

facet_wrap(~traits, scales = "free") +

theme(legend.position = "none")

p <- ggboxplot(gather_data, x = "Sex", y = "Measurement",

fill = "traits",

facet.by = "traits", scales = "free")

p + stat_compare_means(method = "t.test", label.x.npc = "middle", hjust = 0.5, vjust = 0.5, family = "serif", size=7) +

theme(legend.position = "none",

strip.text = element_text(

size = 20),

text=element_text(family= "serif", size=20))

# Show results p-value

results <- results %>% arrange(p_value)

print(results)

write.csv(results,

file = "C:/Users/analorena/Desktop/t_test_results.csv",

row.names = FALSE)

data_wild <- data %>%

na.omit()

data_wild

data_wild$where <- "Contemporary"

data_mu <- data[is.na(data$head), ]

data_mu

data_mu$where <- "Historical"

dat_combine <- rbind(data_mu, data_wild)

t_test_female <- t.test(dat_combine[dat_combine$where == "Historical" & dat_combine$sex == "Female", 10],

dat_combine[dat_combine$where == "Contemporary" & dat_combine$sex == "Female", 10],

var.equal = TRUE)

t_test_female

t_test_male <- t.test(dat_combine[dat_combine$where == "Historical" & dat_combine$sex == "Male", 10],

dat_combine[dat_combine$where == "Contemporary" & dat_combine$sex == "Male", 10],

var.equal = TRUE)

t_test_male

# Boxplot (forearm) Museum vs Wild

morpho_vars <- c("forearm")

data_museum_wild <- dat_combine %>%

dplyr::select(all_of(morpho_vars), sex, where)

colnames(data_museum_wild) <- c("Forearm", "Sex", "Origin")

# Boxplot with Times New Roman

ggplot(data_museum_wild, aes(x = Origin, y = Forearm, fill = Origin)) +

geom_boxplot(alpha = 0.9) +

labs(

title = "Boxplot Forearm: Museum vs Wild",

x = "Specimen Source",

y = "Forearm Length (mm)"

) +

scale_fill_manual(values = c("Historical" = "#619CFF", "Contemporary" = "#F8766D")) +

theme_minimal(base_size = 21) +

theme(

text = element_text(family = "serif", size = 22),

axis.text.x = element_text(size = 25),

axis.text.y = element_text(size = 22),

axis.title.x = element_text(size = 24),

axis.title.y = element_text(size = 24),

legend.position = "none"

)

- **PCA, dendogram, correlations**

library(ggplot2)

library(dplyr)

library(dendextend)

library(tibble)

# Load the data

data <- read.csv(file_path)

# Filter individuals with complete morphometric data

complete_data <- data %>%

filter(!is.na(head),

!is.na(body),

!is.na(leg),

!is.na(ear),

!is.na(weight),

!is.na(forearm))

### Store sex and morphometric variables separately

sex <- complete_data$sex

colnames(complete_data)

morpho_vars <- complete_data[, c("head", "body", "leg", "ear", "weight", "forearm")]

### Perform PCA

pca_result <- prcomp(morpho_vars, scale. = TRUE)

# PCA plot with custom colors (salmon for females, blue for males)

sex <- complete_data$sex

pca_df <- data.frame(PC1 = pca_result$x[,1], PC2 = pca_result$x[,2])

pca_df$sex <- sex

pca_df$Color <- ifelse(pca_df$sex == "Female", "salmon", "blue")

ggplot(pca_df, aes(x = PC1, y = PC2, color = Color)) +

geom_point(size = 3) +

scale_color_identity(guide = "legend",

labels = c("Female", "Male"),

breaks = c("salmon", "blue"),

name = "Sex") +

labs(title = "PCA of D. rotundus Morphometric Data",

x = paste0("PC1 (", round(summary(pca_result)$importance[2,1]*100, 1), "%)"),

y = paste0("PC2 (", round(summary(pca_result)$importance[2,2]*100, 1), "%)")) +

theme_minimal()

### Hierarchical clustering (Euclidean distance + Ward's method)

dist_matrix <- dist(scale(morpho_vars))

hc <- hclust(dist_matrix, method = "ward.D2")

# Dendrogram colored by sex

dend <- as.dendrogram(hc)

labels(dend) <- complete_data$location

sex <- complete_data$sex

label_colors <- ifelse(sex == "Female", "salmon", "blue")

dend <- dend %>%

set("labels_col", label_colors) %>%

set("labels_cex", 0.8)

# Plot

plot(dend, main = "Dendrogram colored by sex (Female = salmon, Male = blue)")

legend("topright", legend = c("Female", "Male"), fill = c("salmon", "blue"), border = NA)

### Correlations between variables

cor_matrix <- cor(morpho_vars[, c("head", "body", "leg", "ear", "weight", "forearm")])

# Correlation matrix

round(cor_matrix, 2)

# Specific correlation tests

cor_ear_forearm <- cor.test(morpho_vars$ear, morpho_vars$forearm)

cor_forearm_weight <- cor.test(morpho_vars$forearm, morpho_vars$weight)

cor_body_weight <- cor.test(morpho_vars$body, morpho_vars$weight)

# Print test results

cor_ear_forearm

cor_forearm_weight

cor_body_weight

- **Outliers**

library(ggplot2)

library(dplyr)

library(readr)

# Load the data

data <- read.csv(file_path)

# Inspect the data

str(data)

summary(data)

# Remove rows with missing values for key variables

clean_data <- data %>%

filter(!is.na(forearm), !is.na(sex))

# Convert sex to a factor

clean_data$sex <- as.factor(clean_data$sex)

#Boxplot with outliers

ggplot(clean_data, aes(x = sex, y = forearm, fill = sex)) +

geom_boxplot(alpha = 0.7, outlier.colour = "red", outlier.shape = 16, outlier.size = 2) +

scale_fill_manual(values = c("Male" = "#1f78b4", "Female" = "orange")) +

labs(title = "Forearm Length by Sex", y = "Forearm Length (mm)") +

theme_minimal()

# Identify outliers by group (sex)

get_outliers <- function(data, variable, group) {

data %>%

group_by({{ group }}) %>%

mutate(

Q1 = quantile({{ variable }}, 0.25, na.rm = TRUE),

Q3 = quantile({{ variable }}, 0.75, na.rm = TRUE),

IQR = Q3 - Q1,

Lower = Q1 - 1.5 * IQR,

Upper = Q3 + 1.5 * IQR,

is_outlier = {{ variable }} < Lower | {{ variable }} > Upper

) %>%

filter(is_outlier) %>%

select({{ group }}, {{ variable }})

}

# Get outliers

outliers <- get_outliers(clean_data, forearm, sex)

print(outliers)

# Add an ID column (if not present)

clean_data$ID <- 1:nrow(clean_data)

# Mark outliers

clean_data <- clean_data %>%

group_by(sex) %>%

mutate(

Q1 = quantile(forearm, 0.25, na.rm = TRUE),

Q3 = quantile(forearm, 0.75, na.rm = TRUE),

IQR = Q3 - Q1,

Lower = Q1 - 1.5 * IQR,

Upper = Q3 + 1.5 * IQR,

is_outlier = forearm < Lower | forearm > Upper

)

# Identify outliers in forearm length grouped by locality

outliers_by_locality <- clean_data %>%

group_by(location) %>%

mutate(

Q1 = quantile(forearm, 0.25, na.rm = TRUE),

Q3 = quantile(forearm, 0.75, na.rm = TRUE),

IQR = Q3 - Q1,

Lower = Q1 - 1.5 * IQR,

Upper = Q3 + 1.5 * IQR,

is_outlier = forearm < Lower | forearm > Upper

) %>%

filter(is_outlier) %>%

select(location, sex, forearm)

# View the outliers

print(outliers_by_locality)

theme_minimal()

# Export the outliers

write_csv(outliers_by_locality, "///")

#Plot outliers

ggplot(clean_data, aes(x = location, y = forearm, fill = sex)) +

geom_boxplot(outlier.shape = NA, alpha = 0.7) +

geom_point(data = filter(clean_data, is_outlier), aes(color = sex), size = 2) +

theme_minimal() +

theme(axis.text.x = element_text(angle = 45, hjust = 1)) +

labs(title = "Forearm Length Outliers by Locality", y = "Forearm Length (mm)")

**MANOVA TEST**

# Load the dataset

file_path <- "C:/Users/analorena/Documents/Dimorphism paper/S1 Table.csv"

manova_model <- manova(cbind(head, body, tibia, ear, weight, forearm) ~ sex, data = complete_data)

summary(manova_model, test = "Wilks")

colnames(complete_data)

# Run MANOVA with correct variable names

manova_model <- manova(cbind(head, body, leg, ear, weight, forearm) ~ sex, data = complete_data)

# View summary with Wilks' Lambda

summary(manova_model, test = "Wilks")

# convert sex as factor

complete_data$sex <- as.factor(complete_data$sex)

# adjust MANOVA

manova_fit <- manova(cbind(head, body, leg, ear, weight, forearm) ~ sex, data = complete_data)

# summary

summary(manova_fit, test = "Pillai")

summary(manova_fit, test = "Wilks")
